# The Supposedly Innocuous Eye Worm: Or, What Could Make You Put Chili in Your Eye?

**DOI:** 10.4269/ajtmh.21-0319

**Published:** 2021-09-13

**Authors:** Luzia Veletzky, Ghyslain Mombo-Ngoma, Michael Ramharter

**Affiliations:** ^1^ Department for Tropical Medicine, Bernhard-Nocht-Institute for Tropical Medicine & I. Department of Medicine, University Medical Center Hamburg-Eppendorf, Hamburg, Germany;; ^2^ Centre de Recherches Médicales de Lambaréné, Gabon;; ^3^ German Center for Infection Research (DZIF), Partner Site Hamburg-Lübeck-Borstel-Riems, Germany

I was perplexed. Did I understand correctly or had I misinterpreted what the woman had told me? Was it maybe a special expression I didn’t understand?

I had just asked the last question, finishing the questionnaire with, “And did you use any treatment against the eye worm?”

The interviewee responded, “Yes, if you have an eye worm, you need to put chili in your eye. That will chase the worm away.”

We were sitting in a small rural village in Gabon, surrounded by lush Central African rainforest that was a vivid contrast to the heavy, dark-blue rain-filled clouds piling up in the sky. It was, as usual, a very hot day, and the Gabonese dampness was making its presence known—a precursor of the upcoming rain that makes every thought exhausting.

Startled, I asked if I had understood the woman’s response correctly. She confirmed, laughing, saying, “Well we have nothing else to treat that worm, so what shall we do? It will chase the worm away and if he comes back you have to repeat the treatment.” Still perplexed, I noted “piment,” which is French for chili pepper, in the study questionnaire as eye worm treatment.

Later, when we discussed this scenario among our team members, our local colleagues were as surprised as I. They had not heard of this treatment option. We all knew about attempts of eye worm removal using old syringes or bamboo sticks, with varying degrees of success; but, the reported application of chili into the eye left us wondering. Was this a singular habit by this woman or a general accepted treatment method?

Gabonese chili is, in fact, very strong; I can bear only small quantities of it in my food without starting to cough and cry. I did not even want to think about coming close to it with my eyes.

The days went by and we continued our field survey, querying about symptoms, history of eye worm, and applied treatments. Despite the fact that loiasis and its most prominent symptom—the “eye worm”—have been known for centuries, the disease is still commonly considered of minor clinical significance and is often not treated.

In the following weeks, many participants shared their treatment of choice against the eye worm, leaving us stunned with each new substance reported. We learned that next to chili, garlic, lemon, and onion juice were very commonly used.

In one village, a witty elderly woman shared with us that, yes, indeed, these ointments chase the worm away, but that there are indigenous plants with juice that actually dissolve the worm when applied.

We were also lucky to meet a traditional healer, well known in the area for her crafts. She showed us the leaves she would cook for hours and then use the extract for eye worm treatment. Because the recipe was a well-kept family secret, we were not allowed to see the plants from which she took the leaves. However, she also kept an old needle, in case a patient preferred direct worm removal.

For the next several months, we became acquainted with the various treatments. Nevertheless, I was still shocked when one man in his 40s told me that he had dropped petrol into his eye in the hope of getting rid of the worm; another man detailed how he had done the same with his own urine. Fortunately, they reported no persistent damage, which was not true for others. One participant had a clear opacity in his left eye that developed after he administered menthol to his eye to chase away the eye worm. Ever since, he could see objects only hazily.

Although most affected people reported they were kept from their work by eye worm migration, some individual stories stuck in my head. One young man told me he had tried several eye drops from the pharmacy that were supposed to help, but didn’t. He had a job in a saw mill, where he was handling the computers running the machines. Several times per year he was unable to work because the worm would make his sight blurry in the affected eye—a condition that made his work with these saws much too dangerous.

When I told these stories to colleagues working on loiasis, their first reaction usually was: “Yeah, well these treatments won’t work. Maybe the caused irritation makes the eye worm disappear quicker into the subcutaneous tissue, but they won’t treat the infection, so why do you bother?

Indeed, I never argued for the efficacy of these treatments, which need to be assessed before they can be judged. My point was how irritating and disturbing an eye worm migration must be if affected patients actually applied these substances to their eyes. Treatments used varied from patients desperately trying all kind of liquids, to old recipes for extracts, which require several hours of preparation. This shows the role of the symptom in the society, where the disease is well known and the majority of adults are affected at least once during their lifetime.

In the region, medical treatment is difficult to access, but these people are risking their sight to get temporary relief from the worm. All these stories of treatment attempts from so many different people are a driving argument that loiasis is a serious infection. The eye worm is not an interesting sign that is great for case reports. It is a pathogen that needs to be taken seriously and treated appropriately. Since the first woman told me about the chili treatment, one question still remains: What would make me deliberately put chili, my own urine, or fuel into my eye?

I have not found the answer yet.

